# What are the risk factors of colonoscopic perforation?

**DOI:** 10.1186/1471-230X-9-71

**Published:** 2009-09-24

**Authors:** Varut Lohsiriwat, Sasithorn Sujarittanakarn, Thawatchai Akaraviputh, Narong Lertakyamanee, Darin Lohsiriwat, Udom Kachinthorn

**Affiliations:** 1 Siriraj GI Endoscopy center, Department of Surgery, Faculty of Medicine Siriraj Hospital, Mahidol University, Bangkok 10700, Thailand; 2 Siriraj GI Endoscopy center, Department of Medicine, Faculty of Medicine Siriraj Hospital, Mahidol University, Bangkok 10700, Thailand

## Abstract

**Background:**

Knowledge of the factors influencing colonoscopic perforation (CP) is of decisive importance, especially with regard to the avoidance or minimization of the perforations. The aim of this study was to determine the incidence and risk factors of CP in one of the endoscopic training centers accredited by the World Gastroenterology Organization.

**Methods:**

The prospectively collected data were reviewed of all patients undergoing either colonoscopy or flexible sigmoidoscopy at the Faculty of Medicine Siriraj Hospital, Bangkok, Thailand between January 2005 and July 2008. The incidence of CP was evaluated. Eight independent patient-, endoscopist- and endoscopy-related variables were analyzed by a multivariate model to determine their association with CP.

**Results:**

Over a 3.5-year period, 10,124 endoscopic procedures of the colon (8,987 colonoscopies and 1,137 flexible sigmoidoscopies) were performed. There were 15 colonic perforations (0.15%). Colonoscopy had a slightly higher risk of CP than flexible sigmoidoscopy (OR 1.77, 95%CI 0.23-13.51; p = 1.0). Patient gender, emergency endoscopy, anesthetic method, and the specialty or experience of the endoscopist were not significantly predictive of CP rate. In multivariate analysis, patient age of over 75 years (OR = 6.24, 95%CI 2.26-17.26; p < 0.001) and therapeutic endoscopy (OR = 2.98, 95%CI 1.08-8.23; p = 0.036) were the only two independent risk factors for CP.

**Conclusion:**

The incidence of CP in this study was 0.15%. Patient age of over 75 years and therapeutic colonoscopy were two important risk factors for CP.

## Background

Colonoscopy is a common procedure used for the diagnosis and treatment of a wide range of colorectal diseases. There are an increasing number of patients undergoing endoscopic examination of the colon and rectum for various purposes such as screening and surveillance of colorectal cancer. One of the most serious complications of colonoscopy is endoscopic perforation of the colon, which has been reported as between 0.03% and 0.7% [1,2]. Although colonoscopic perforation (CP) occurs rarely, it can be associated with high mortality and morbidity rates. Recently, we have reported a CP rate of 0.09% from 17,357 endoscopic procedures between 1999 and 2007 in our institute. This was associated with 13% mortality and 53% morbidity [3]. Recently, two extensive reviews of the outcomes following CP by Iqbal [4] and Teoh [5] showed a mortality rate of 7-26% and a morbidity rate of 37-49%, together with a 38% rate of intestinal stoma formation.

Knowledge of the factors influencing CP is of decisive importance, especially with regard to the avoidance or minimization of such a serious complication. However, there is a paucity of literature on identification of the risk factors associated with CP and the results are controversial. For instance, some investigators have suggested that advanced age of patients and endoscopy performed by a trainee increased the risk of CP [6,7], whereas other investigators have found that these factors were not predictive of a higher risk of CP [8-11]. The aim of this study was to determine the incidence and risk factors of CP in a single large endoscopic training center.

## Methods

### Patients

We carried out an analysis of all patients who underwent either colonoscopy or flexible sigmoidoscopy at the Siriraj GI Endoscopy center, Faculty of Medicine Siriraj Hospital, Mahidol University, Bangkok, Thailand between January 2005 and July 2008. This endoscopic training center was accredited by the World Gastroenterology Organization (WGO) in 2006. Patients younger than 15 years were excluded from this study. Data were prospectively collected in the hospital's computer database, including data on a 30-day follow-up period. The primary end points of the study were endoscopic perforation of the colon. Risk factors for such a complication were then analyzed. The study was approved by the Institutional Ethics Committee.

### Endoscopic procedure

All patients undergoing colonoscopic examination received mechanical bowel preparation using either 2 liters of polyethylene glycol or 90 ml of sodium phosphate, whereas patients undergoing flexible sigmoidoscopic examination received mechanical bowel preparation or rectal enema. In the case of an emergency setting, defined as non-scheduled endoscopic examination of the colon for acute colonic conditions such as lower gastrointestinal bleeding, patients would undergo the aforementioned protocol of bowel preparation if possible.

Endoscopic examination was performed with or without sedation depending on the patient's requirement and the endoscopist's preference. In the sedation group, intravenous propofol and fentanyl were administrated by an anesthesiologist. These drugs are well suited to colonoscopy due to their rapid onset of action, and short duration. Other sedative drugs, such as benzodiazepams, were rarely used in our unit. In the non-sedation group, there were no analgesics given before, during or after the procedure. Endoscopy was performed by either a gastroenterologist or a general surgeon. The extent of colon visualization while performing sigmoidoscopy is up to the splenic flexure, or about 60 cm. from the anal verge. Any training fellows were involved in colonic endoscopies under the close supervision of a well-experienced endoscopist.

### Definition of colonoscopic perforation

Colonoscopic perforation was considered to be present if any of the followings was observed: visualization of extra-intestinal structure during the endoscopic examination, presence of pneumoperitoneum or retroperitoneal gas with signs of peritonitis after the procedure, and intraoperative finding of a perforated colon.

### Statistical analysis

Eight independent patient-, endoscopist-, and endoscopy-related variables were analyzed. Patient-related variables were age and gender. Endoscopist-related variables were the specialty of the endoscopist (gastroenterologist or surgeon), and whether a training fellow was involved in the procedure. Endoscopy-related variables were procedure (colonoscopy or flexible sigmoidoscopy), purpose of the procedure (diagnostic or therapeutic), endoscopic setting (emergency or elective), and whether the examination was conducted with or without sedation.

All data were prepared and compiled using SPSS computer program (version 11.0 for Windows). The Mann-Whitney U test and Chi-square test were used to compare data between CP group and non-CP group. The univariate relation between each independent variable and colonoscopic perforation was tested and the odds ratio (OR) with 95% confidence intervals (CI) for each variable was determined. Any significant variables in the univariate analysis were included in a multivariate model of logistic regression. A P-value of less than 0.05 was considered statistically significant. The statistics have been checked and analyzed by a medical statistician in our faculty.

## Results

Over a 3.5-year period, 10,124 endoscopic procedures of the colon (8,987 colonoscopies and 1,137 flexible sigmoidoscopies) were performed. Patients had an average age of 59 years (SD = 14.5, range 15-103) and 55 percent were female. Three main indications for endoscopic examination of the colon and rectum in this series were clinical suspicion of colorectal neoplasia, anemia or hematochezia work-up, and screening and surveillance of colorectal cancer. Forty-two percent of the patients underwent colonoscopy under sedation (n = 4,202). Therapeutic endoscopy and emergency endoscopy were performed in 24% (n = 2,385) and 0.7% (n = 75), respectively. Of therapeutic endoscopy, snared polypectomies were performed in 1589 cases (67%). Acute lower gastrointestinal bleeding was the major indication for emergency endoscopy.

Fifteen colonic perforations (0.15%) were identified; 14 from colonoscopy and one from flexible sigmoidoscopy. None occurred during emergency endoscopy. All the perforations were identified during or shortly after the procedure; extra-intestinal tissue was observed during the procedure in 13 cases (87%) and the others developed peritonitis within 24 hours after the procedure with the presence of pneumoperitoneum detected on a CT scan. The most common site of CP was in the sigmoid colon (n = 12, 80%), and followed by the transverse colon (n = 2, 13%) and the benign stricture site of ileorectal anastomosis (n = 1, 7%). Of these patients, three (20%) had concomitant colorectal cancer. All CP patients underwent surgical management. Types of operation included primary suture of the perforation in 5 patients (33%), resection and primary anastomosis in 4 patients (27%), and resection without anastomosis in 6 patients (40%). There were 2 deaths (women at the age of 76 and 83), accounting for 13% of CP patients and 0.02% of total colonic endoscopy.

Based on the endoscopic and intraoperative findings, the most common mechanism of perforation was determined to be direct trauma from the shaft of the endoscope (n = 6, 40%), and followed by trauma from the tip of the endoscope (n = 5, 33%). Details of all clinical features of the perforation are shown in table 1.

**Table 1:** Details of patients with colonoscopic perforation (CP)

Age, Sex	Endoscopic procedure	Endoscopist	Perforated site	Possible mechanism of CP
77 M*	D (FS)	Staff	Sigmoid colon	SHAFT
76 F	D	Staff	Sigmoid colon	SHAFT
88 M*	D	Staff	Sigmoid colon	SHAFT
76 F*	D	Trainee	Sigmoid colon	SHAFT
34 M*	D	Trainee	Transverse colon	SHAFT
64 F*	D	Staff	Sigmoid colon	TIP
81 M	D	Trainee	Sigmoid colon	TIP
79 M	D	Trainee	Sigmoid colon	Accidental entry to a large diverticulum
27 F	T (hot biopsy)	Trainee	Sigmoid colon	SHAFT
46 M*	T (hot biopsy)	Staff	Sigmoid colon	TIP
64 M*	T (hot biopsy)	Staff	Sigmoid colon	TIP
79 F*	T (cold biopsy)	Staff	Transverse colon	TIP
37 F*	T (snare polypectomy)	Trainee	Sigmoid colon	Transmural biopsy
53 F*	T (snare polypectomy)	Staff	Sigmoid colon	Transmural biopsy
59 F	T (pneumatic dilatation of benign stricture)	Staff	Stricture site	Over-stretching of the benign stricture

* Previously reported in Lohsiriwat et al. [3]

Note: One perforation occurred during flexible sigmoidoscopy (FS).

Abbreviation: D = Diagnostic endoscopy, T = Therapeutic endoscopy, TIP = mechanical trauma from the tip of scope, SHAFT = mechanical trauma from the shaft of scope

Colonoscopy had a slightly higher incidence of CP than flexible sigmoidoscopy (0.16% vs 0.09%, OR 1.77, 95%CI 0.23-13.51; p = 1.0). The incidence of CP following therapeutic endoscopies was significantly higher than that following diagnostic endoscopies (0.29% vs 0.1%; p = 0.035). Trainee endoscopists were involved in 29% of the endoscopies performed (n = 2,938). These trainee-performed endoscopies resulted in 6 out of the total of 15 perforations, accounting for 0.2% incidence of CP in the trainee-performed cases. However, trainee endoscopists did not significantly increase rate of CP (OR 1.63, 95%CI 0.58-4.59; p = 0.35). Patient gender, emergency endoscopy, anesthetic method, and the specialty or experience of the endoscopist were not significantly predictive of CP rate. All the patient-, endoscopist-, and endoscopy-related variables and their association with CP were analyzed by the univariate and multivariate analysis (Table 2). In multivariate analysis, patient age of over 75 years (OR = 6.24, 95%CI 2.26-17.26; p < 0.001) and therapeutic endoscopy (OR = 2.98, 95%CI 1.08-8.23; p = 0.036) were the only two independent risk factors for CP.

**Table 2:** Univariate and multivariate analysis of risk factors for colonoscopic perforation

Variable	Univariate analysis		Multivariate analysis	
	OR (95%CI)	P-value	OR (95%CI)	P-value
Age over 75	6.05 (2.19-16.7)	<0.001	6.24 (2.3-17.3)	<0.001
Procedure (T vs D)	2.85 (1.03-7.85)	0.035	2.98 (1.08-8.2)	0.036
Endoscopy (C vs FS)	1.77 (0.23-13.5)	1.00		
Trainee	1.63 (0.58-4.59)	0.35		
Sedation	1.61 (0.58-4.45)	0.35		
Endoscopist (G vs S)	1.08 (0.39-2.99)	0.88		
Male	1.07 (0.39-2.96)	0.89		

Abbreviation: T = Therapeutic endoscopy, D = Diagnostic endoscopy, C = Colonoscopy, FS = Flexible sigmoidoscopy, G = Gastroentrologist, S = Surgeon

## Discussion

In this high-volume single endoscopic training center, we found that the incidence of perforation from colonoscopy was 1.6 per 1000 procedures (0.16%) and from sigmoidoscopy was 0.9 per 1000 procedures (0.09%). There were 8 perforations among the diagnostic colonoscopies (1.0 per 1000 procedures) and 7 perforations among the therapeutic colonoscopies (2.9 per 1000 procedures). These incidences were 2-3 times higher than those of our previous review, in which the incidence of perforation from colonoscopy was 0.1% and from sigmoidoscopy was 0.03% [3]. One possible explanation of this finding is that the number of colonoscopies and endoscopic procedures has remarkably increased over the last few years, and more colonoscopies are being performed by trainees in our institute. It is also plausible there are an increasing number of patients receiving sedation during colonoscopy, which may thus affects the endoscopist's perception of alarming pain experienced by the patients. However, the CP incidence in our present study is still comparable to that reported in other larger series (sample size > 30,000 cases) [6,12,13]. Based on the present study, both univariate and multivariate analysis showed that therapeutic endoscopy and patient age of over 75 years were two important risk factors for CP.

Performing therapeutic intervention during colonoscopy has been shown to increase the risk of CP in several studies [2,14]. The increased likelihood of CP in therapeutic endoscopy is because the perforation during therapeutic colonoscopy can occur not only through mechanisms that are similar to those seen for diagnostic colonoscopy (mechanical injury or barotrauma), but also through the fact that endoscopic interventions *per se *can cause perforation. Several investigators have suggested that some endoscopic interventions are associated with an increased CP rate, including polypectomy for polyps larger than 20 mm [15], endoscopic submucosal resection for colorectal neoplasia [16], and pneumatic dilatation for anastomotic colonic stricture [17]. Regarding the clinical presentations of CP, a recent multicenter review by Teoh and colleagues showed that the perforations occurring during therapeutic colonoscopies were significantly smaller in size than those occurring during diagnostic colonoscopies, and that the patients presented later [5].

Although some investigators reported that older age was not predictive of an increased risk of CP [8,9], in the present study, there was an approximately six-fold rise in the CP rate in patients over 75 years. This finding was consistent with that of Gatto and colleagues [6], in which CP rate climbed fourfold to 0.3% in patients with age over 75. A possible explanation for this finding is that the elderly might have a declining colonic wall mechanical strength which is partly a consequence of changes in the collagen structure [18]. Perhaps, an increasing number of diverticular diseases in the elderly may contribute to a higher rate of CP because an endoscopist could inadvertently push a scope through a large diverticulum (see Table 1.), or snare an inverted diverticulum simulating a polyp [19]. Moreover, there is a greater frequency of abnormal colorectal findings detected in the elderly which require endoscopic intervention [20].

A few population-based studies have evaluated the independent effects of patient, endoscopist and setting factors on CP. In the US Medicare cohort of 39,286 colonoscopies between 1991 and 1998, increased age and increased co-morbidity were predictive of increased CP rate [6]. A more recent and larger population-based study in Canada has revealed that advanced age, patient with high co-morbidity and endoscopic polypectomy were independently associated with CP [21]. The later study otherwise confirmed our findings from the WGO endoscopic training center in Thailand that older age and therapeutic endoscopy are two major risk factors of CP.

A tendency toward increased likelihood of CP in our study has also been found in patients undergoing colonoscopy as opposed to sigmoidoscopy, trainee involvement, and endoscopy under intravenous sedation, although none of these groups reached statistical significance.

Similar to our findings, a population-based study in the United States showed that the risk of perforation after colonoscopy was approximately double that after sigmoidoscopy [6]. A review of over 30,000 patients in the Netherlands also showed that the relative risk ratio of colonoscopic and sigmoidoscopic procedures for perforations was four [12]. Another study at the Mayo Clinic, by Anderson and co-workers [8], found an incidence of 1.9 perforations per 1000 colonoscopies and 0.4 perforations per 1000 sigmoidoscopies.

Colonoscopy by a training fellow or a less-experienced endoscopist is believed to increase rate of CP. However, most published studies have been unable to demonstrate any significant impact of trainee endoscopist on the increased rates of CP [8,10,11]. One possible explanation of this observation is that staff endoscopists may accept more challenging cases than trainees. Another possible reason is that trainees may be more cautious about performing colonoscopy as they are still in the supervised process of learning. In 2001, the Society of American Gastrointestinal Endoscopic Surgeons (SAGES) Colonoscopy Study Outcome Group reported that there was no association between experience of the endoscopist and complications [10]. However, a minimum of 50 prior colonoscopies and 100 annual colonoscopies could be associated with a significant improvement in the rate of colonoscopic completion. Unlike SAGES study, a smaller retrospective study, by Galandiuk and Ahmad [7], showed that the complication rate of colonoscopy performed by trainees was significantly higher than that performed by staff endoscopists.

Colonoscopy is generally performed with intravenous sedation and analgesia because it is sometimes painful and causes patient discomfort. Several studies have suggested that sedation in colonoscopy is associated with a higher percentage of complete examinations [22,23]. Colonoscopy under intravenous sedation appears to increase risk of CP in the present study. It is possible that an endoscopist has difficulty in recognizing over-stretching of the bowel wall in the deep-sedated patients, thus leading to mechanical injury to the colon. Perhaps, endoscopists manipulate the scope non-forcefully and more gently in patients without sedation.

Based on our study, there was no significant difference in the perforation rate between procedures carried out by gastroenterologists (0.15%) and ones by surgeons (0.14%). This finding was confirmed by other investigators [5]. Patient gender was also not predictive of CP in the present study; however, some authors have shown that female patients were at greater risk of the perforation [8]. Interestingly, Saunders and co-workers demonstrated that women had a greater colonic length and a more mobile transverse colon [24]. Other risk factors for CP reported in the literature could include a history of diverticular disease or previous intra-abdominal surgery [25].

Although a relatively small number of potential risk factors for CP were recorded and analyzed in the present study, they are among the fundamental parameters commonly used for patient risk prediction [20]. Future research should investigate further possible factors influencing CP, such as the quality of bowel preparation, previous intra-abdominal or pelvic operation, prior pelvic radiotherapy, a previous history of intra-abdominal or pelvic infection, diverticular disease, presence of colonic malignancy and co-morbidities. Meanwhile, given the rarity of the perforation incidence, data collection from several endoscopic centers, in order to increase the sample and event size as well as analysis of other potential risk factors, would give physicians more insights into what increases the incidence of CP and how to avoid or minimize it.

## Conclusion

According to this study, the incidence rate of CP was 0.15%. Patient age of over 75 years and therapeutic colonoscopy were two important risk factors for CP. Our findings have indicated that special precautions should be made during therapeutic endoscopy and while performing colonoscopic examination in the elderly, particularly in patients over 75 years. Non-invasive investigation of the colon such as CT colonography, if applicable, might be considered in such advanced age patients.

## Abbreviations

CI: confidence intervals; CP: colonoscopic perforation; OR: odds ratio; WGO: World Gastroenterology Organization

## Competing interests

The authors declare that they have no competing interests.

## Authors' contributions

VL was the principal investigator who participated in research design, analyzed the data, and prepared the manuscript. SS and TA contributed to acquisition and analysis of data. NL, DL and UK conceived of the study, participated in its design and coordination, and helped to draft the manuscript. All authors read and approved the final manuscript.

## Pre-publication history

The pre-publication history for this paper can be accessed here:

http://www.biomedcentral.com/1471-230X/9/71/prepub
